# Complete Mitochondrial Genome of *Phytophthora nicotianae* and Identification of Molecular Markers for the Oomycetes

**DOI:** 10.3389/fmicb.2017.01484

**Published:** 2017-08-08

**Authors:** Xiaolong Yuan, Chao Feng, Zhongfeng Zhang, Chengsheng Zhang

**Affiliations:** ^1^Tobacco Research Institute of Chinese Academy of Agricultural Sciences Qingdao, China; ^2^Tobacco Pest Integrated Management Key Laboratory of China Tobacco Qingdao, China

**Keywords:** *Phytophthora nicotianae*, mitochondrial genome, comparative genomics, phylogenetic relationship, molecular markers

## Abstract

*Phytophthora nicotianae* is one of the most destructive plant pathogens affecting a variety of plants, causing black shank of tobacco, among several other devastating diseases. Herein, we assembled the mitochondrial genome of *P. nicotianae* and analyzed its gene content and genome structure, performed comparative mitochondrial genomics analysis, and assessed phylogenetic relationships among oomycetes species. The circular mitogenome is 37,561 bp long, with 38 protein-coding genes, 25 transfer RNA (tRNA) genes, and 2 ribosomal RNA genes (rrnl and rrns). The mitochondrial genome showed a biased A/T usage versus G/C. The overall gene content and size of the *P. nicotianae* mitogenome are identical to those of other published *Phytophthora* mitogenomes. Interestingly, collinearity analysis using an existing ∼10 k inversion region (including 11 genes and 8 tRNAs) revealed that *Phytophthora andina*, *Phytophthora infestans*, *Phytophthora mirabilis*, *Phytophthora ipomoeae*, and *Phytophthora phaseoli* differed from *Phytophthora nicotianae*, *Phytophthora sojae*, *Phytophthora ramorum*, and *Phytophthora polonica*. Moreover, inverted repeat regions were found to be absent among species of the Peronosporales when compared with species from the Pythiales and Saprolegniales. A phylogenomic investigation based on 29 protein-coding genes demonstrated that *Phytophthora* is monophyletic, and placed *P. nicotianae* close to the clade including *P. mirabilis*, *P. ipomoeae*, *P. andina*, *P. infestans*, and *P. phaseoli*. Furthermore, we discovered six new candidate DNA molecular markers (*rpl6*, *atp8*, *nad11*, *rps2*, *rps3*, and *rps4*) based on these mitogenomes that would be suitable for species identification in the oomycetes, which have the same identification level as the whole mitogenome and ribosomal DNA sequences. These new molecular markers can not only provide a quick preview of the species without mitogenome information, but will also help to gain better understanding of the oomycetes pathogens and developing treatment or monitoring strategies.

## Introduction

*Phytophthora nicotianae* is an important destructive plant pathogen, which causes root, stem, and crown rots, as well as fruit and foliar blights, in over 301 agronomic and horticultural plants (Panabières et al., 2016). For example, it can cause black shank in cultivated tobacco and significantly decrease the crop yield, leading to catastrophic economic losses (van Jaarsveld et al., 2002; Falcón-Rodríguez et al., 2011). This pathogen can cause disease by releasing oospores or chlamydospores into the soil. The morphological characteristics of the sporangia of *P. nicotianae* are an ovoid/spherical shape with an average size of 46.4 × 37.8 pm, and the average size of the chlamydospores is approximately 19–42 pm (Blaya et al., 2015). Further research has revealed multiple physiological races of *P. nicotianae* (races 0, 1, 2, and 3) worldwide (Apple, 1962). Races 0 and 1 have also been identified in the tobacco-growing areas of China. *P. nicotianae* has been extensively studied from several aspects to date, including its pathology and comprehensive strategies for the prevention of devastating outbreaks (Csinos, 1999; Judelson and Roberts, 1996; Bowers and Locke, 2004). For example, strategies recommended for managing black shank include applying chemicals and using resistant tobacco varieties (Speight, 1994; Qu et al., 2016). Moreover, comparative morphological and molecular characterization studies of *P. nicotianae* have also been conducted (Smith et al., 1986; Cooke et al., 2000). Phylogenetic analysis revealed that *P. nicotianae* is closely related to diatoms and brown algae, which is contrast to the results obtained through morphological and physiological identification (Cooke et al., 2000). In general, molecular phylogenetic studies to determine the relationships among *Phytophthora* species and related members of the oomycetes mainly based on the internal transcribed spacer (ITS) region of nuclear ribosomal DNA (rDNA) have been used to explore the relationships, especially for distinguishing morphologically similar species among *Phytophthora* and some other species at the genus level (Crawford et al., 1996). Blair et al. (2008) conducted a comprehensive analysis of the relationships among 82 *Phytophthora* species utilizing seven loci from available databases. However, most of these previous molecular studies have explored the relationships among *Phytophthora* species using only one or a few genetic loci (Blair et al., 2008).

Recently, the mitogenome, which represents the entire mitochondrial genome including both the coding and non-coding regions, was used for resolving evolutionary relationships between organisms whose morphological and biochemical characters are often limited or difficult to obtain because of small genome size, high copy numbers, and rapid evolutionary rates (Chen and Hebert, 1999). Consequently, the number of oomycete mitogenomes (*Phytophthora*, *Pythium*, *Thraustotheca*, *Saprolegnia*, and *Achlya*) that have been partially or completely sequenced has been steadily increasing in recent years (Grayburn et al., 2004; Avila-Adame et al., 2006; Martin et al., 2007; O’Brien et al., 2014; Tangphatsornruang et al., 2016). These published sequences have the potential to speed up the development of the classification, evolution, genetics, breeding, and engineering of the corresponding mycetes. With respect to *Phytophthora* species, eight complete mitogenomes have been sequenced thus far. The mitochondrial genome of *Phytophthora infestans* was used to investigate its evolution and population genetics (Gavino and Fry, 2002). In addition, comparative genomic analysis was performed among *P. infestans*, *P. ramorum*, and *P. sojae* mitochondrial genomes to illustrate their evolutionary relationships (Martin et al., 2007). Although the nuclear genome of *P. nicotianae* has been sequenced to illustrate its infection mechanisms and coevolution with host plants (Liu et al., 2016), knowledge of its complete mitogenome is still lacking. In addition, the mitogenome and mitochondrial genes can be considered effective markers for evolutionary studies because their evolutionary rates are 5–10 times higher than those of nuclear DNA (Brown et al., 1979). To expand the pool of available molecular markers from the oomycetes mitochondrial genome, it is necessary to establish the phylogeny of the genus *Phytophthora* using available mitogenomic data and identify new phylogenetically informative molecular markers that can be used to identify a greater number of *Phytophthora* species.

Accordingly, the objectives of this study were to sequence the complete mitogenome of *P. nicotianae*, provide a thorough description of its genome features, discuss the phylogenetic relationships and evolutionary traits among species of the oomycetes based on the sequenced mitogenomes, and determine novel molecular markers that would be suitable for identification studies. The current study will provide valuable reference information for gaining further understanding of mitogenome evolution in related species.

## Materials and Methods

### Culture and Collection

The highly virulent *P. nicotianae* strain JM1 was isolated from infected leaves of *Nicotiana benthamiana* in Jimo, Qingdao, Shandong Province. The mycelium was cultivated at 25°C on clarified V8 agar (von Broembsen and Deacon, 1996). The cultivated mycelium was washed using sterilized distilled water and then dried with filter paper before use.

### DNA Sequencing and Assembly

Total genomic DNA from the mycelium was extracted using a Qiagen DNEasy Plant Mini Kit according to the manufacturer’s instructions (Qiagen, Valencia, CA, United States). The sequencing libraries were generated using IlluminaTruseqTM DNA Sample Preparation Kit (Illumina, San Diego, CA, United States) following the manufacturer’s instructions. First, 2 μg genomic DNA was fragmented using the S220-series ultrasonicator (Covaris, Woburn, MA, United States). Then, exonuclease/polymerase was added to convert the overhangs into blunt ends. The 3′ ends of DNA fragments were adenylated and ligated to Illumina PE adapter oligonucleotides. Fragments of approximately 650 bp were then separated with AMPure Beads (Agencourt Bioscience, Beverly, MA, United States). The products were purified using QIAquick PCR Purification Kit (Qiagen) and amplified using Illumina PCR Primer for 10 cycles. After purification, the PCR products were quantified using the Agilent Bioanalyzer 2100 system. Finally, the library was sequenced with the Illumina HiSeq 2000 sequencing system. The overall 2G raw reads were quality trimmed, and adapters were removed using the FastQC software with default parameters (version 0.11.5) (Andrews, 2017). Reads with hits to the mitochondrial genomes from previously published *Phytophthora* studies were extracted using a local blast search (1e5). These reads were then assembled with the CLC Genomics workbench with default parameters (version 4.7.1). Next, conserved contigs (occupying 75% of the total length of the mitogenome) were used to extend the remaining sequences using PRICE with default parameters (version 3.0) (Ruby et al., 2013). Finally, the topological ring of this mitogenome was detected by Bandage with default parameters^1^ (Wick et al., 2015).

### Identification and Annotation of the Mitochondrial Genome

The mitogenome was annotated with DOGMA^2^ (Wyman et al., 2004), followed by application of NCBI ORF finder^3^. Transfer RNA (tRNA) genes were identified by tRNAscan-SE (version 1.23) (Schattner et al., 2005). 23S and 16S rRNA genes were annotated based on similarity of the reference *Phytophthora* mitochondrial rRNA sequence. The circular mitochondrial genome map was drawn using OGDRAW^4^ (Lohse et al., 2013). The relative synonymous codon usage of protein-coding genes (PCGs) was calculated using CodonW (Peden, 1999). The complete mitochondrial genome of *P. nicotianae* is available under GenBank accession number KY851301.

### Analyses of Gene Evolutionary Rates and Molecular Marker Selection

To estimate the nature of evolutionary selection on the common genes shared in the Peronosporales, the genes involved in ATP-synthase complex subunits (complex IV and complex III subunits), electron transport complex I subunits, and the large and small subunits of ribosomal proteins were chosen to calculate the ratio of non-synonymous (dN) to synonymous (dS) substitutions. These genes were aligned using MEGA 6.0 according to codons (parameters: Gap opening penalty:10; Gap extension penalty:0.2; Delay divergent cutoff:30%). Then, the dN/dS ratio was calculated using DnaSP ver. 5 (Librado and Rozas, 2009). Usually, the synonymous sites (dS) are saturated in the closely related species, and thus the non-synonymous substitution sites (dN) are suitable for measuring evolutionary rates. Therefore, the dN values and their standard deviations were calculated. Then the genes in higher dN value (0.02 < dN < 0.5) and wider standard deviation (higher than the 50% of the 29 genes) are more likely to provide better resolution in lower level phylogenies and discriminate between short distance species like subspecies. To further confirm the identification of these candidate genes, we conducted phylogenetic analyses using candidate genes after conducting model tests. The rDNA results were used as reference for comparison with the mitogenome results to select the final gene markers for species identification of oomycetes.

### Comparative Analysis of the Mitogenome

The mitogenome of *P. nicotianae* was compared to previously published mitogenomes of the oomycetes. For the comparison of gene content and orders, the genetic context of each mitogenome was extracted using a perl script^5^, and visualized in a vector graph. For synteny comparison, the mitogenomes of 13 species in the oomycetes were downloaded from GenBank, and together with the *P. nicotianae* (Supplementary Table S1) mitogenome completed in the present study were parsed through pairwise blast results among members of the oomycetes using Mauve software with default parameters (Darling et al., 2004). The invert repeat structure was detected using the blastn program against the sequence itself and visualized in Circoletto^6^ (Darzentas, 2010).

### Phylogenetic Analyses

To infer the phylogenetic position of *P. nicotianae*, we chose 13 oomycete mitogenome sequences available in GenBank. We conducted separate phylogenetic analyses for individual genes and the combined mitochondrial DNA datasets. The dataset of 29 PCGs present in the 14 mitochondrial genomes (including species of *Phytophthora*, *Pythium*, *Thraustotheca*, *Saprolegnia*, and *Achlya*) were used for phylogenetic analysis based on both maximum likelihood (ML) and Bayesian inference (BI) methods. Individual genes of 14 species were aligned using MEGA 6.0 one by one and then formed the final alignment. The nucleotide substitution model was selected using jModelTest (V2.1.4) (Posada, 2008). ML analysis was performed using RAxML version 8.1.2 (Stamatakis, 2014) with the GTR + I + G model. Bootstrap analysis was conducted with 1000 replications in the ML method. Bayesian analyses were performed using MrBayes (Ronquist et al., 2012) and run for 1,000,000 generations, with the initial 20% of trees discarded as burn-in. Moreover, phylogenetic analyses were also conducted with rDNA sequences from same taxa using BI approaches under the optimized nucleotide evolutionary models (Supplementary Table S2). For these parameters, “p-inv” represents the proportion of invariant sites among the selected sequences. “Gammaeshape” represents a two-parameter family of continuous probability distributions and “Kappa” represents the substitution parameters among the selected sequences.

## Results

### Genome Size and Organization

**FIGURE 1:**
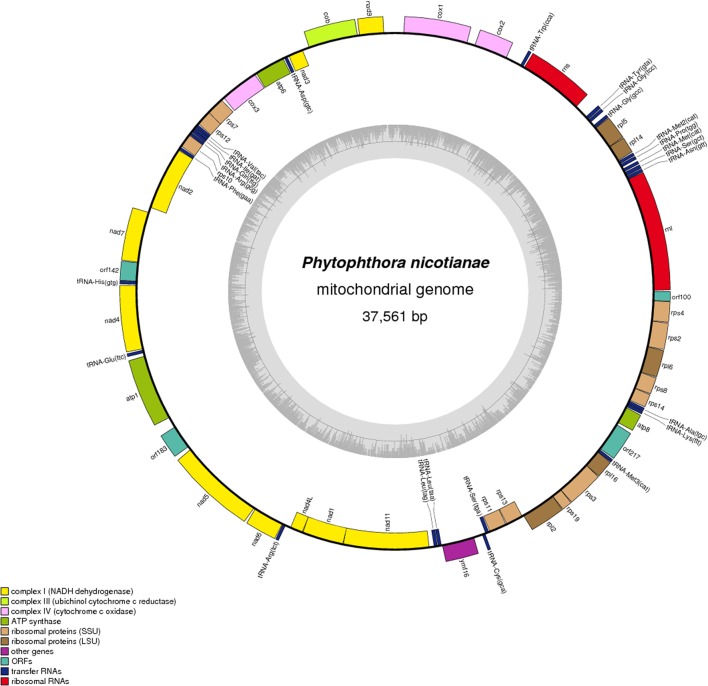
Circular map of the mitochondrial genome of *Phytophthora nicotianae.* Protein coding genes, tRNA and rRNA are shown on the outer ring. Genes encoded on both strands. The inner ring shows GC density.

The complete sequence of the *P. nicotianae* mitochondrial genome was mapped as a 37,561-bp long circular molecule (with approximately 1000-fold coverage) (**Figure 1**). Approximately 91.02% of the genome comprises the coding region. We identified 38 PCGs, 25 tRNA genes, and 2 rRNA genes (*rrnL* and *rrnS*), which are located on both strands. All the PCGs start with the typical ATG codon and terminate with either TAA or TAG. No intron or overlap was found in these genes. The *P. nicotianae* mitochondrial genome contains 1,901 bp within tRNA genes, with a G + C content of 37.82%. Individual tRNAs range in length from 71 bp [trn-Cys (GCA)] to 91 bp [trn-Ser1 (GCT)] in length. The two rRNA genes in the mitochondrial genome, rrnL and rrnS, are 2,656 bp and 1,503 bp in length, with an G + C content of 33.25% and 35.46%, spectively (**Tables 1**,**2**).

**Table 1:** General features of *Phytophthora nicotianae* mitochondrial genome.

Genome features	Value
Genome size (bp)	37561
G + C content (%)	21.88
No. of protein-coding genes	38
A + T content of protein-coding genes (%)	79.53
Structural proteins coding exons (%)	74.89
No. of rRNAs/tRNAs	2/25
A + T content of RNA genes (%)	64.49
rRNAs + tRNAs (%)	16.13
Coding regions (%)	91.02
Intergenic regions (%)	8.98
No. of introns	0
No. of intronic ORFs	0
Introns (%)	0

**Table 2:** Gene organization of the *P. nicotianae* mitochondrial genome.

Gene	Position		Length		Codon	
	From	To	nt	aa	Start	Stop
*Rnl*	24	2679	2656	–	–	–
*trn-Asn (GTT)*	2685	2758	74	–	–	–
*trn-Ser1 (GCT)*	2769	2859	91	–	–	–
*trn-Met1 (CAT)*	2879	2950	72	–	–	–
*trn-Pro (TGG)*	2996	3070	75	–	–	–
*trn-Met2 (CAT)*	3084	3155	72	–	–	–
*rpl14*	3173	3544	372	123	ATG	TAA
*rpl5*	3551	4084	534	178	ATG	TAA
*trn-Gly1 (GCC)*	4091	4164	74	–	–	–
*trn-Gly2 (TCC)*	4264	4337	74	–	–	–
*trn-Tyr (GTA)*	4350	4433	84	–	–	–
*rns*	4758	6260	1503	–	–	–
*trn-Trp (CCA)*	6295	6366	72	–	–	–
*cox2*	6739	7515	777	259	ATG	TAA
*cox1*	7710	9188	1479	493	ATG	TAA
*nad9*	9648	10214	567	189	ATG	TAA
*cob*	10262	11410	1149	383	ATG	TAA
*nad3*	11573	11926	354	118	ATG	TAA
*trn-Asp (GTC)*	11962	12037	76	–	–	–
*atp6*	12062	12781	720	240	ATG	TAA
*cox3*	12809	13726	918	306	ATG	TAA
*rps7*	13782	14210	429	143	ATG	ATT
*rps12*	14185	14565	381	127	ATG	TAA
*trn-Val (TAC)*	14582	14654	73	–	–	–
*trn-Ile (TAC)*	14657	14730	74	–	–	–
*trn-Gln (TTG)*	14731	14804	74	–	–	–
*trn-Arg1 (GCG)*	14813	14888	76	–	–	–
*rps10*	14892	15218	327	109	ATG	TAA
*trn-Phe (GAA)*	15237	15310	74	–	–	–
*nad2*	15317	16810	1494	498	ATG	TAA
*nad7*	16927	18105	1179	393	ATG	TAA
*orf142*	18117	18545	429	143	ATG	TAA
*trn-His (GTG)*	18549	18621	73	–	–	–
*nad4*	18644	20119	1476	492	ATG	TAA
*trn-Glu (TTC)*	20147	20218	72	–	–	–
*atp1*	20298	21827	1530	510	ATG	TAA
*orf183*	22084	22635	552	184	ATG	TAA
*nad5*	22724	24718	1995	665	ATG	TAA
*nad6*	24762	25472	711	237	ATG	TAA
*trn-Arg2 (TCT)*	25486	25558	73	–	–	–
*nad4L*	25677	25979	303	101	ATG	TAA
*nad1*	25982	26962	981	327	ATG	TAA
*nad11*	26959	28968	2010	670	ATG	TGA
*trn-Leu1 (TAG)*	29075	29159	85	–	–	–
*trn-Leu2 (TAA)*	29169	29252	84	–	–	–
*ymf16*	29275	30018	744	248	ATG	TAA
*trn-Cys (GCA)*	30226	30296	71	–	–	–
*trn-Ser2 (TGA)*	30308	30392	85	–	–	–
*rps11*	30411	30827	417	139	ATG	TAA
*rps13*	30840	31253	414	138	ATG	TAA
*rpl2*	31282	32085	804	268	ATG	TAA
*rps19*	32089	32325	237	79	ATG	TAA
*rps3*	32329	33144	816	272	ATG	TAA
*rpl16*	33147	33551	405	135	ATG	TAA
*trn-Met3 (CAT)*	33554	33627	74	–	–	–
*orf217*	33649	34320	672	224	ATG	TAA
*atp8*	34382	34774	393	131	ATG	TAA
*trn-Lys (TTT)*	34795	34869	75	–	–	–
*trn-Ala (TGC)*	34872	34945	74	–	–	–
*rps14*	34963	35262	300	100	ATG	TAA
*rps8*	35271	35651	381	127	ATG	TAA
*rpl6*	35662	36267	606	202	ATG	TAA
*rps2*	36278	36871	594	198	ATG	TAA
*rps4*	36880	37341	462	154	ATG	TAA
*orf100*	37344	37559	216	72	ATG	TAA

**FIGURE 2:**
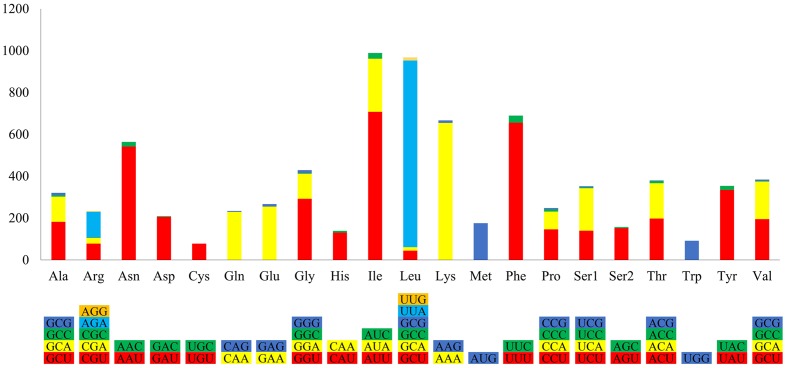
Relative synonymous codon usage of the mitochondrial genome in *P. nicotianae.* Colorful columns represent the codon usage of the genes in *P. nicotianae*. The height of columns represents the frequency of utilization of a certain codon.

The *P. nicotianae* mitochondrial genome shows very strong A/T bias in nucleotide composition relative to the G + C content (A + T content of 78.12%), which is typical of oomycete mitochondrial genomes (**Table 1**). Analysis of the codon usage showed that Ile, Leu, and Phe are the most frequently encoded amino acids in the *P. nicotianae* mitochondrial genome. Hence, ATT (Ile), TTA (Leu), and TTT (Phe) are the most frequent codons, which contribute to the high A + T content observed in this mitochondrial genome (**Figure 2**).

### Gene Content and Structure Comparison

**FIGURE 3:**
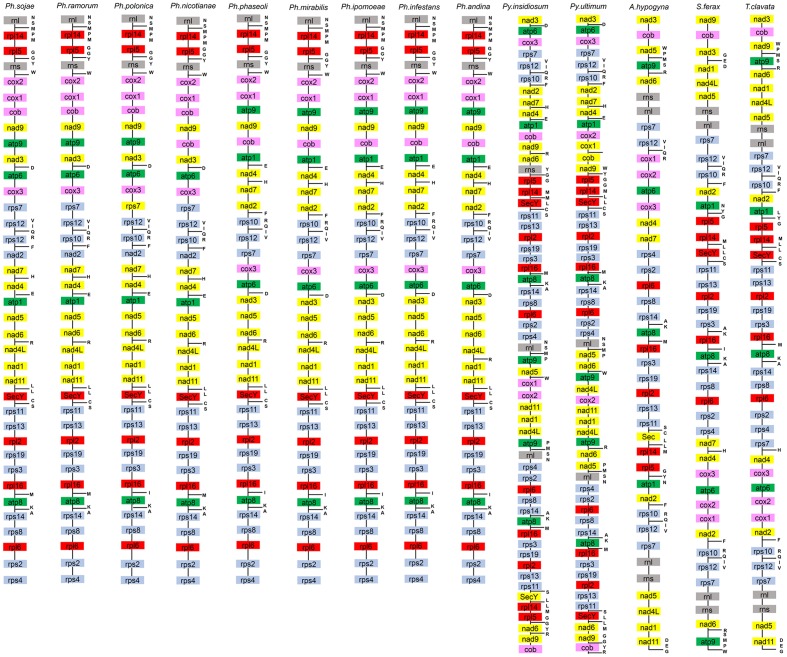
Mitochondrial gene orders of 14 species from the Oomycetes. Gene orders and contents of 14 species are listed from top to bottom. Genes in different colors represent different functions. tRNA which can comprise amino acids are listed on the right of gene contents.

The gene content of *P. nicotianae* was compared to those of 13 other species in the oomycetes (including species from the three orders Peronosporales, Pythiales, and Saprolegniales), with available mitochondrial genomes information. The genome size of the *P. nicotianae* mitogenome is 37,561 bp, which is similar to that of other *Phytophthora* species (e.g., 37,957 bp for *P. infestans* and 37,874 bp for *P. andina*). The gene content of the *P. nicotianae* mitogenome is identical to that of the published *Phytophthora* mitogenomes. They all have 35 common PCGs. Some small differences in gene contents were observed only in open reading frames (ORFs). For example, six ORFs (orf79, orf32, orf142, orf244, orf217, and orf100) were found in *P. mirabilis*, *P. andina*, *P. infestans*, *P. ipomoeae*, and *P. phaseoli*, whereas *P. sojae*, *P. ramorum*, *P. nicotianae*, and *P. polonica* contained 12, 9, 4, and 6 predicted ORFs, respectively. Overall, this comparative study demonstrated that the gene composition and orders are highly conserved among species in *Phytophthora* mitogenomes. This phenomenon was also observed in the Pythiales mitogenomes, whereas major differences in gene content were observed for the Saprolegniales sequences. The genome size in the Pythiales was greater than that for species from the Peronosporales because of duplication events that occurred in the *nad6*, *cob*, *nad9*, *atp9*, *rpl5*, *rpl14*, *rnl*, *rps4*, *rps2*, *rpl6*, *rps8*, *rps14*, *atp8*, *rpl16*, *rps3*, *rps19*, *rpl2*, *rps13*, *rps11*, and *SecY* genes. In addition, the gene content in the Peronosporales was compared with that of the Saprolegniales, which showed that the genes *rnl*, *rns*, *nad5*, *rps7*, and *rps12* were doubled in Saprolegniales species (**Figure 3**).

**FIGURE 4:**
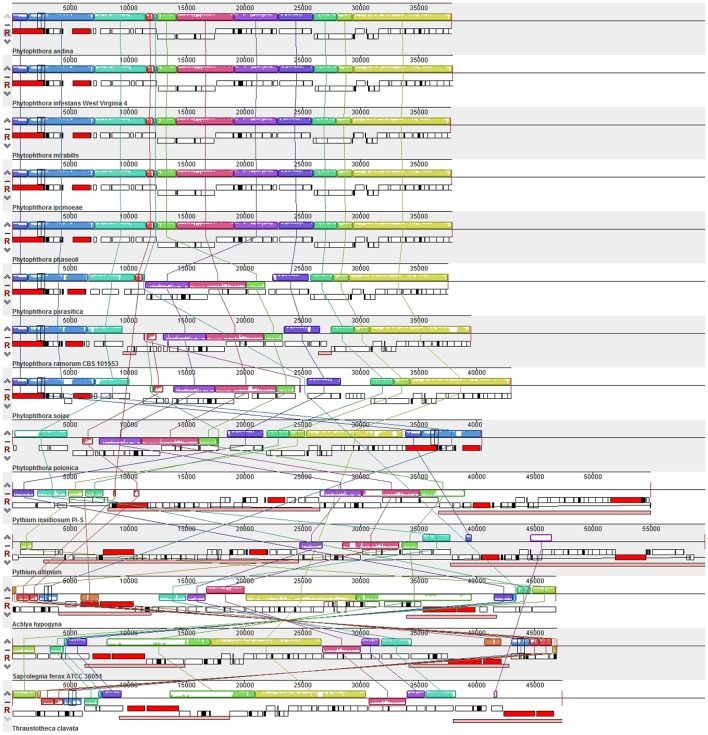
Collinearity analysis of the genome structures of 14 mitogenomes from the Oomycetes. Collinear blocks among 14 species are identified by MAUVE. Links between these collinear blocks are indicated by the thin colored lines.

**FIGURE 5:**
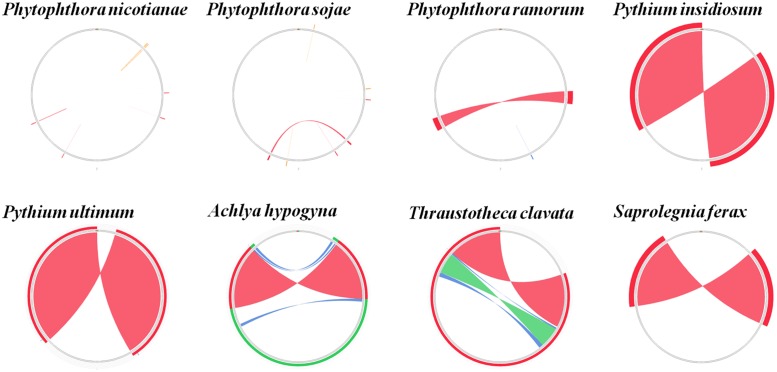
Comparison of inverted repeat regions in oomycetes species. There were no repeat regions in *P. nicotianae* and *P. sojae*. Repeat regions of 1.1 kb repeat regions were found in *P. amorum*. Reverted repeat regions of 18.3, 22, 7.7, 9.4, and 8.6 kb reverted repeat regions were found in *P. insidiosum*, *P. ultimum*, *A. hypogyna*, *T. clavata*, and *S. ferax*. These repeat regions were detected by blasting against the sequences themselves. The colors represent alignment scores and can reflect he similarity of sequences: red (≥200), green (50–80,) and blue (40–50).

Comparison of the genome structures of the 14 mitogenomes from the Oomycetes showed that, despite their evolutionary distance, the genomes shared 12 modules (syntenic regions) in the Peronosporales. Interestingly, collinearity analysis using a ∼10 k inversion region (including 11 genes: *nad3*, *atp6*, *cox3*, *rps7*, *rps12*, *rps10*, *nad2*, *nad7*, *orf142*, *nad4*, and *atp1*; and 8 tRNAs: tRNA-Asp, tRNA-Val, tRNA-Ile, tRNA-Gln, tRNA-Arg, tRNA-Phe, tRNA-His, tRNA-Glu) revealed that *P. andina*, *P. infestans*, *P. mirabilis*, *P. ipomoeae*, and *P. phaseoli* differed from *P. nicotianae*, *P. sojae*, *P. ramorum*, and *P. polonica*. Moreover, our study showed that, unlike species from the Pythiales and Saprolegniales, inverted repeat (IR) regions were absent among species in the Peronosporales. The species in the Pythiales and the Saprolegniales have large IR regions, which contributed to expanding their genome sizes. The Pythiales species shared six modules, whereas the Saprolegniales species had 14 gene modules (**Figure 4**). Synteny comparison revealed that the IR regions were absent from species in the Peronosporales. However, according to blastn against the sequence itself, a shorter repeat region of about 2,300 bp was found in *P. ramorum*. The IR regions in *Pythium insidiosum* and *Pythium ultimum* were 36,530 and 43,898 bp, occupying 66.43 and 73.54% of the whole sequences, respectively. The repeat regions in the Saprolegniales ranged from 15,542 to 18,848 bp (**Figure 5**).

### Evolutionary Rates of Common Genes in the Peronosporales

**FIGURE 6:**
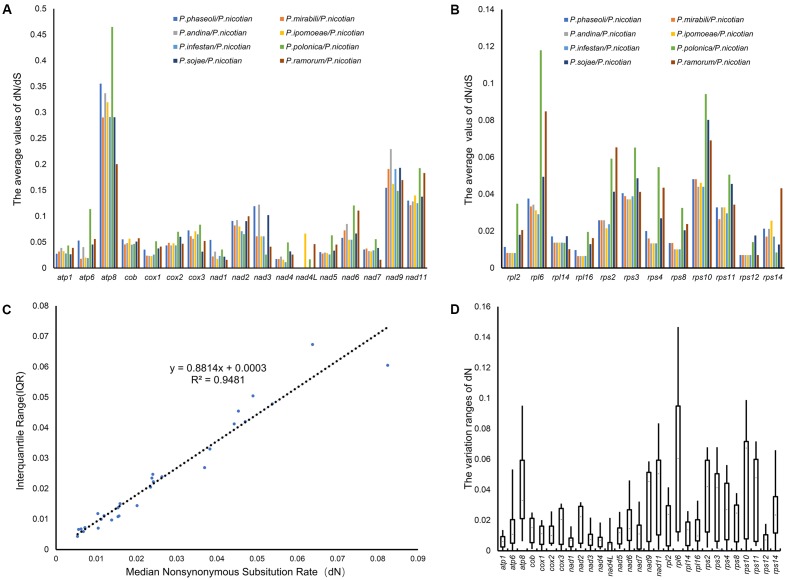
Evolutionary rates of common genes in the Peronosporales. **(A,B)** dN/ds ratio of pair-wise comparison among the nine species in the Peronosporales. **(C)** dN values and their standard deviations. **(D)** The Box-plot of dN values.

To determine the nature of the evolutionary selection pressure in Peronosporales species, we calculated the evolutionary rate of 29 genes, and simultaneously chose candidate genes that could serve as molecular markers for identification based on variation of their evolutionary rates. The analysis showed that the dN/dS ratio changed from 0.016 to 0.448, suggesting that these genes underwent negative selection pressure in the process of evolution. The highest dN/dS ratio was found in *atp8* (0.448), followed by *rps10* (0.314) and *rpl6* (0.273). However, the lowest of dN/dS ratios were detected for *rps12*, *nad7*, and *nad4L* (0.016, 0.033, and 0.034, respectively). The overall value of dN/dS in ribosomal genes was higher than that for the other genes (**Figures 6A,B**). In generally, the synonymous mutation rate (dN) and its variation range (i.e., standard deviation) can be used to determine the genetic evolutionary rate, especially for genes that are under purification selection. Therefore, the value of dN, and the correlations between dN and their interquartile ranges (IQRs) for individual genes were calculated. The value of dN ranged from 0.006 to 0.064, meeting the criteria for DNA barcoding (dN < 0.5) (Janouškovec et al., 2013). dN and IQR were found to be highly correlated (*R*^2^ = 0.95) (**Figure 6C**). Genes with higher dN values and wider standard deviations are more likely to provide better resolution in lower-level phylogenies for discrimination between more closely related taxa such as subspecies. To find suitable target molecular markers, we sorted these genes into several bins according to their dN values, (high dN value range from 0.02 to 0.06: *rpl6, rps10, atp8, nad11, rps11, rps2, rps3, nad9, rps14* and *rps4*; medium dN value range from 0.01 to 0.02: *rps8, rpl2, nad6, nad2, atp6, cox3, rpl16, cob, rpl14, nad7, cox2, nad5* and *cox1*; low dN value range from 0.006 to 0.01: *nad3, nad4, rps12, nad1, atp1* and *nad4L*). Therefore, the genes in higher dN value (0.02 < dN < 0.5) were chosen as the candidates. Meanwhile, the genes with wider standard deviation (higher than the 50% of the 29 genes) were also selected, including the genes *atp6, atp8, cox3, nad2, nad6, nad9, nad11, rpl2, rpl6, rps2, rps3, rps4, rps8, rps10, rps11* and *rps14.* Overall, *rpl6, rps10, atp8, nad11, rps11, rps2, rps3, nad9*, and *rps4* were chosen as suitable molecular markers for the identification of species in the Peronosporales (**Figure 6D**). Based on these results, *rpl6*, *rps10*, *atp8*, *nad11*, *rps11*, *rps2*, *rps3*, *nad9*, and *rps4* were chosen as the molecular markers for the identification of species in the Peronosporales.

### Phylogenomic Analyses

**FIGURE 7:**
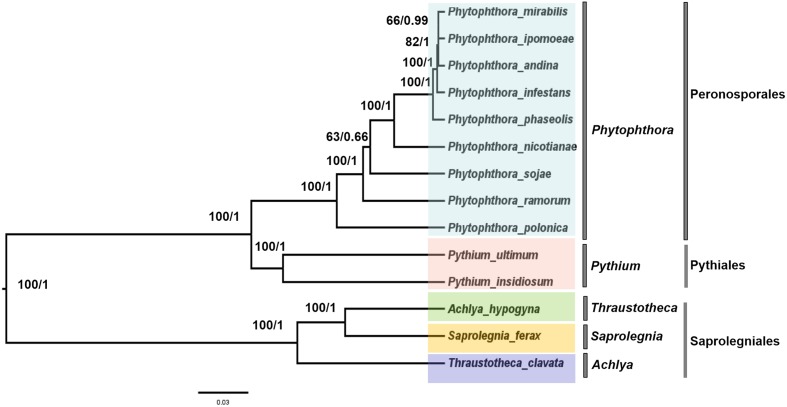
Phylogenetic analysis of oomycetes species based on 29 common mitochondrial DNA sequences. ML and Bayesian trees were constructed based on 24,244 nucleic acids. Bootstrap values (first value) and Bayesian inference (second value) are presented above the branches.

**FIGURE 8:**
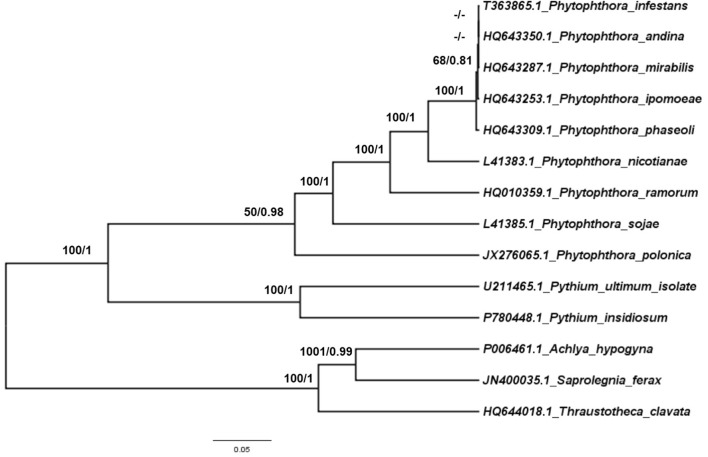
Phylogenetic analysis of oomycetes species based on rDNA mitochondrial DNA sequences. ML and Bayesian trees were constructed based on rDNA mitochondrial DNA sequences. Bootstrap values (first value) and Bayesian inference (second value) are presented above the branches. Numbers at nodes are likelihood bootstrap values > 50% and posterior probabilities > 0.60 and maximum are shown.

To trace the evolutionary position of the *P. nicotianae* specimen analyzed, we reconstructed phylogenies within the Oomycetes on an aligned data matrix that included 14 taxa and 29 mitochondrial PCGs with a total length of 24,244 bp. The ML and Bayesian trees revealed consistent phylogenetic relationships. Our results supported the monophyly of all orders studied herein. The phylogenomic investigation demonstrated that *Phytophthora* is monophyletic, and *P. nicotianae* was positioned close to the clade including *P. mirabilis*, *P. ipomoeae*, *P. andina*, *P. infestans*, and *P. phaseoli* with high bootstrap support (100%), which was confirmed in the Bayesian topology (value of 1). The species in the Pythiales were sister to all other species in the Peronosporales, and the species in the Saprolegniales clustered in a clade at the root of the Oomycetes tree (**Figure 7**). Moreover, the phylogenetic relationships based on rDNA in our analyses corresponded with the mitogenome results (**Figure 8**). In addition, for further confirmation, we conducted separate phylogenetic analyses for the candidate marker genes indicated above based on their optimization models. Our results identified six new DNA molecular markers (*rpl6*, *atp8*, *nad11*, *rps2*, *rps3*, and *rps4*) for oomycetes species identification based on these mitogenomes, which showed the same identification ability as the whole mitogenome and rDNA sequences (**Supplementary Figures S1**–**S6**).

## Discussion

### Mitogenome Content and Features of Oomycetes Species

We assembled the complete mitochondrial genomes of *P. nicotianae* and performed a comparative mitochondrial genomic analysis using other previously published oomycete mitochondrial genomes. The comparison illustrated that *P. nicotianae* is highly similar to other *Phytophthora* species with respect to genome size, GC% content, and gene content. However, all the genomes are smaller than those of the related oomycete species in the *Pythiales* (*P. insidiosum*, 54,989 bp; *P. ultimum*, 59,689 bp) (Lévesque et al., 2010; Tangphatsornruang et al., 2016) and in the Saprolegniales (*Saprolegnia ferax*, 46,930 bp; *Achlya hypogyna*, 46,840 bp; *Thraustotheca clavata*, 47382 bp) (O’Brien et al., 2014). All the mitogenomes of *Phytophthora* have a compact gene arrangement, with 35 predicted PCGs, 2 rRNAs, and a set of 25 tRNAs specifying 19 amino acids (Paquin et al., 1997; Avila-Adame et al., 2006; Martin and Richardson, 2007). The overall gene content comparison study demonstrated that the gene components in mitogenomes are highly conserved among species in *Phytophthora.* However, the gene contents of the species in the Pythiales and Saprolegniales are larger than those in the species from the Peronosporales because of duplication events (O’Brien et al., 2014).

### Genome Structure Comparison

Comprehensive comparison of the complete mitochondrial genomes of nine species in the Peronosporales, two species in the Pythiales, and three species in the Saprolegniales was performed to confirm the presence of the genomic rearrangements. Usually, the expansion and contraction of IR regions can cause gene duplications. Our study demonstrated that the IR expansion could have induced more gene duplication and genome expansion events in the Pythiales and Saprolegniales compared with the Peronosporales. In general, there is no IR detected in the genus *Phytophthora*. We found small IR regions of about 2.3 kb in the *P. ramorum* mitochondrial genome, which is consistent with an earlier study (Martin and Richardson, 2007). In contrast, IR regions were found in the Pythiales and Saprolegniales. The IR region can increase the gene dosage of ribosomal components (Bock and Knoop, 2012), and the presence of larger IRs in these genomes might serve to stabilize the genome (Hudspeth et al., 1983). This can be ascribed to the stability of IRs to intramolecular homologous recombination, the only effect of which is to reverse the order of the unique sequences between the arms of the repeat regions (Adelberg and Bergquist, 1972). Among the species in the Oomycetes, the IR is an ancestral feature. However, the reason for the IR loss in the Peronosporales is unclear. Thus, it is proposed that IR regions are not conserved and may not be an essential part of the mitochondrial genome. During their evolution, the species in the Peronosporales might have lost the IR regions in response to tremendous environmental pressure. The presence of IR regions might indicate that they represent hotspots for rearrangement (Martin and Richardson, 2007). For the structure comparison in the Peronosporales, collinearity analysis revealed that *P. andina*, *P. infestans*, *P. mirabilis*, *P. ipomoeae*, and *P. phaseoli* differed from *P. nicotianae*, *P. sojae*, *P. ramorum*, and *P. polonica* over a 12 k inversion region containing 11 genes and 8 tRNAs. The IR regions occurred on different strands. We deduced that this inverted structure is formed during the process of IR loss. Collinearity analysis classified these species into two different groups, consistent with their phylogenetic relationships based on whole mitogenomes. Therefore, analysis of gene structure appears to be a reliable approach to determine phylogenetic relationships in the Oomycetes. Based on the collinearity and phylogenetic relationship analyses, we speculated that the nine species of *Phytophthora* should be divided into two different groups, or perhaps even two genera; however, this hypothesis warrants further in-depth investigation for confirmation.

### Molecular Markers Selection Based on the Mitogenomes

The genus *Phytophthora* includes a large diversity of devastating plant pathogens, which pose serious threats to agriculture and food production (Judelson and Blanco, 2005). Owing to their significant economic importance, molecular genetics and genomics research on *Phytophthora* species has been increasing (Govers and Gijzen, 2006). Individual genes—multiple loci from both the nuclear and mitochondrial genomes—have been used to explore the relationships among morphologically analogous *Phytophthora* species (Crawford et al., 1996). ITS regions have been confirmed as valuable molecular markers to determine evolutionary distance because they contain large number of variable sites (Cooke et al., 2000; Kroon et al., 2004; Feliner and Rosselló, 2007). Blair et al. (2008) presented a genus-wide phylogeny for 82 *Phytophthora* species using 7 of the most informative loci. Recent molecular analyses have substantially increased our understanding of the phylogenetic relationships among *Phytophthora* species (Cooke et al., 2000; Martin and Tooley, 2003). In this study, we present the phylogenetic analysis of rDNA, candidate genes, and whole mitogenomes of 14 species in the Oomycetes from the Pythiales, Saprolegniales, and Peronosporales. Our phylogenetic tree based on whole mitochondrial genomes has thus improved the current understanding regarding evolution of the oomycetes, and has unraveled several mitochondrial DNA genes that are suitable for use as molecular markers in future studies. The whole mitogenome can establish a well-resolved phylogeny of species in the Oomycetes. The multicopy nature of mitochondrial genomes in organisms make them appropriate sources of molecular marker identification. We estimated the evolutionary rates (dN/dS) of common genes among species in the Peronosporales to identify the candidate genes to serve as species identification markers. The results showed that these genes underwent pure selection pressure in the process of evolution. That is to say, the synonymous sites (dS) are saturated among these species and thus non-synonymous substitution sites (dN) are suitable for measuring evolutionary rates. Genes with higher dN values and wider standard deviations are more likely to provide better resolution in lower level phylogenies and discriminate between more closely related taxa such as subspecies. Moreover, we used the rDNA as reference for comparison with the mitogenome results to select the final gene markers for species identification of oomycetes. In other words, the genes showing similar resolution as rDNA and mitochondrial genomes were selected as the new molecular markers. Overall*, rpl6, rps10, atp8, nad11, rps11, rps2, rps3, nad9*, and *rps4* were chosen as suitable molecular markers for the identification of species in the Peronosporales. The newly determined DNA molecular markers from the mitogenome can enrich the species identification for interpreting the evolutionary history of species in the Oomycetes. Thus, in future phylogenetic analyses, these newly determined DNA molecular markers can be used, together with the available nuclear DNA markers, especially for rare species or those from which mitochondrial genomes are hard to retrieve. Moreover, for unsequenced mitochondrial genomes, such molecular markers can provide a quick preview of their likely phylogenetic relationships. These markers can further serve as a useful guide for detecting oomycetes pathogens and developing treatment or monitoring strategies.

## Conclusion

In our study, we presented a comprehensive investigation of the mitochondrial genome of *P. nicotianae.* The phylogeny based on the concatenated mitogenome data supports that *P. nicotianae* belongs to the *Phytophthora.* Comparative analyses suggest that the mitochondrial genome in *Phytophthora* is highly conserved except for an existing unique inversion region. Furthermore, we identified some useful molecular markers for molecular investigations to aid in species delimitation in the Oomycetes.

## Author Contributions

CZ and ZZ conceived and designed the project. XY performed the experiments. XY drafted and revised the manuscript. XY and CF analyzed and interpreted the data. All authors have read and approved the final manuscript.

## Conflict of Interest Statement

The authors declare that the research was conducted in the absence of any commercial or financial relationships that could be construed as a potential conflict of interest.

## Footnotes

1 http://rrwick.github.io/Bandage/

2 http://dogma.ccbb.utexas.edu/

3 http://www.ncbi.nlm.nih.gov/gorf/gorf.html

4 http://ogdraw.mpimp-golm.mpg.de/

5 https://github.com/rayrock01/Extract_gene.pl

6 http://tools.bat.infspire.org/circoletto/

## Supplementary Material

FIGURE S1 Phylogenetic analysis of oomycetes species based on *atp8* gene.

FIGURE S2 Phylogenetic analysis of oomycetes species based on *rpl16* gene.png.

FIGURE S3 Phylogenetic analysis of oomycetes species based on *nad11* gene.

FIGURE S4 Phylogenetic analysis of oomycetes species based on *rps2* gene.

FIGURE S5 Phylogenetic analysis of oomycetes species based on *rps4* gene.

FIGURE S6 Phylogenetic analysis of oomycetes species based on *rps3* gene.
