# Supplementary figures and images for: Complete Mitochondrial Genome of Phytophthora nicotianae and Identification of Molecular Markers for the Oomycetes
Source: Front Microbiol. 2017 Aug 8;8:1484. doi: 10.3389/fmicb.2017.01484 (PMC5550686; doi:10.3389/fmicb.2017.01484)

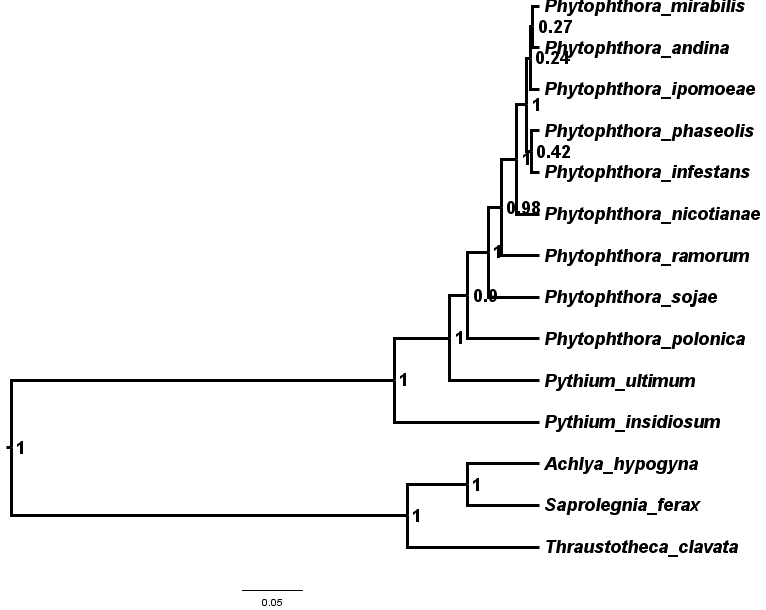

Supplement: FIGURE S1 — Phylogenetic analysis of oomycetes species based on atp8 gene. [file Image_1.PNG]

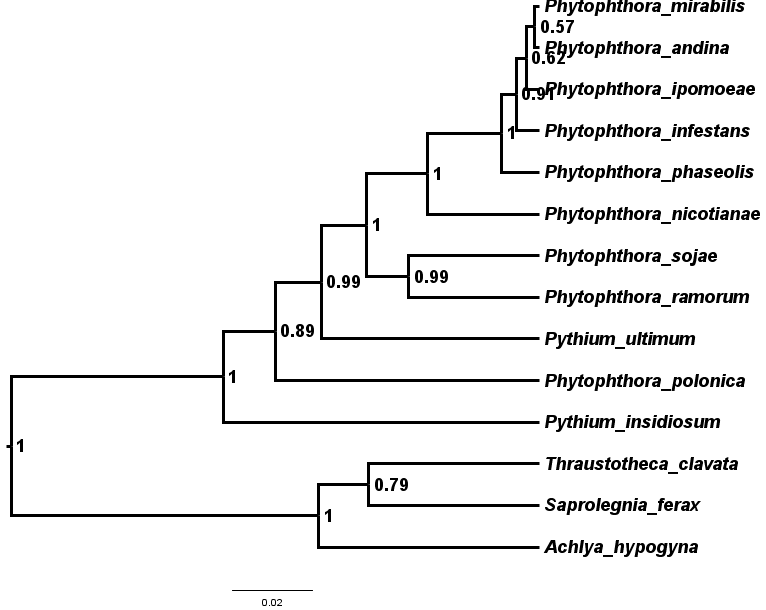

Supplement: FIGURE S2 — Phylogenetic analysis of oomycetes species based on rpl16 gene.png. [file Image_2.PNG]

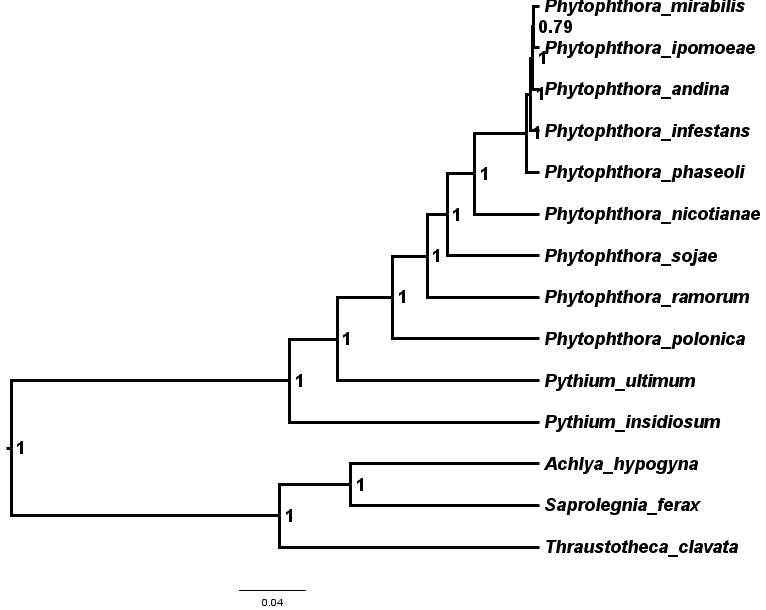

Supplement: FIGURE S3 — Phylogenetic analysis of oomycetes species based on nad11 gene. [file Image_3.PNG]

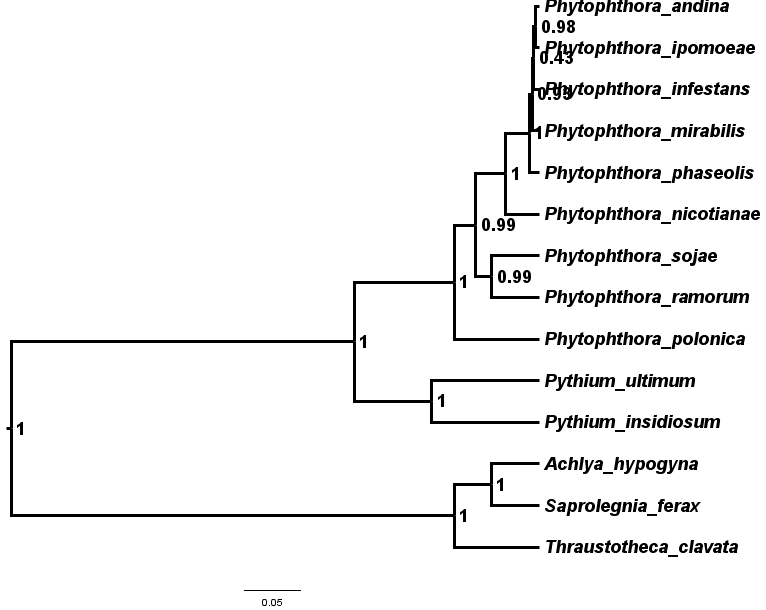

Supplement: FIGURE S4 — Phylogenetic analysis of oomycetes species based on rps2 gene. [file Image_4.PNG]

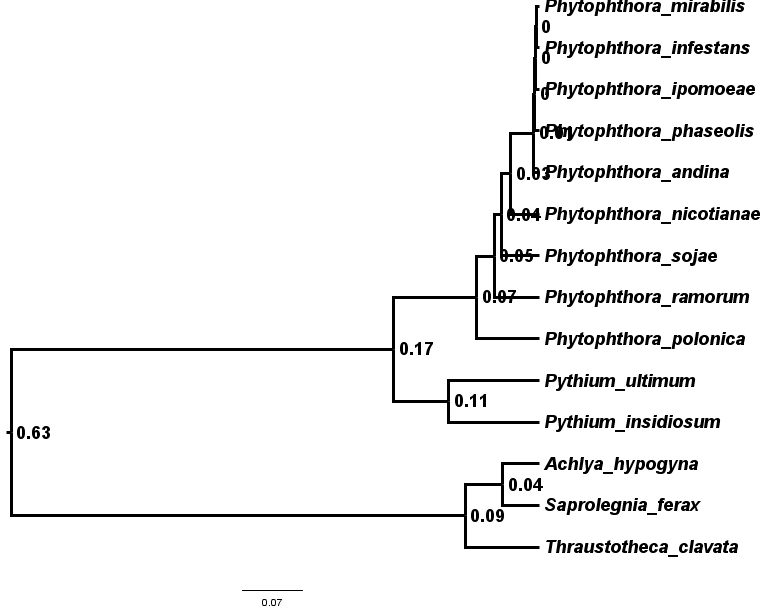

Supplement: FIGURE S5 — Phylogenetic analysis of oomycetes species based on rps4 gene. [file Image_5.PNG]

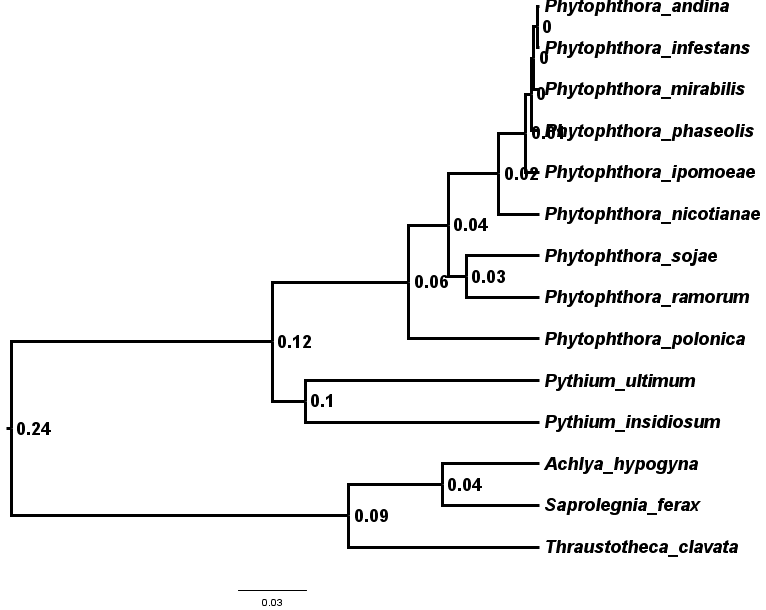

Supplement: FIGURE S6 — Phylogenetic analysis of oomycetes species based on rps3 gene. [file Image_6.PNG]
