# Supplementary material for: Complete Mitochondrial Genome of Phytophthora nicotianae and Identification of Molecular Markers for the Oomycetes
Source: Front Microbiol. 2017 Aug 8;8:1484. doi: 10.3389/fmicb.2017.01484 (PMC5550686; doi:10.3389/fmicb.2017.01484)
Supplement: Supplementary file 7 [file Table_1.DOC]

**Table S1. Mitochondrial genomes used for gene content comparisons and phylogenetic analysis.**

| Species | Genus | Class | Order | Family | Size (bp) | Accession Number |
| --- | --- | --- | --- | --- | --- | --- |
| *Achlya hypogyna* | *Achlya* | Saprolegniaceae | Saprolegniales | Oomycetes | 46,840 | NC022178 |
| *Saprolegnia ferax* | *Saprolegnia* | Saprolegniaceae | Saprolegniales | Oomycetes | 46,930 | AY534144 |
| *Thraustotheca clavata* | *Thraustotheca* | Saprolegniaceae | Saprolegniales | Oomycetes | 47,382 | KF226725 |
| *Pythium insidiosum* | *Pythium* | Pythiaceae | Pythiales | Oomycetes | 54,989 | AP014838 |
| *Pythium ultimum* | *Pythium* | Pythiaceae | Pythiales | Oomycetes | 59,689 | GU138662 |
| *Phytophthora andina* | *Phytophthora* | NA | Peronosporales | Oomycetes | 37,874 | HM590419 |
| *Phytophthora infestans* | *Phytophthora* | NA | Peronosporales | Oomycetes | 37,957 | U17009 |
| *Phytophthora ipomoeae* | *Phytophthora* | NA | Peronosporales | Oomycetes | 37,872 | HM590420 |
| *Phytophthora mirabilis* | *Phytophthora* | NA | Peronosporales | Oomycetes | 37,779 | HM590421 |
| *Phytophthora phaseoli* | *Phytophthora* | NA | Peronosporales | Oomycetes | 37,914 | HM590418 |
| *Phytophthora polonica* | *Phytophthora* | NA | Peronosporales | Oomycetes | 40,467 | KT946598 |
| *Phytophthora ramorum* | *Phytophthora* | NA | Peronosporales | Oomycetes | 39,494 | EU427470 |
| *Phytophthora sojae* | *Phytophthora* | NA | Peronosporales | Oomycetes | 42,977 | DQ832717 |
| *Phytophthora nicotianae* | *Phytophthora* | NA | Peronosporales | Oomycetes | 37,561 | KY851301 |

NA: no related information available in GenBank.
