# Supplementary material for: Complete Mitochondrial Genome of Phytophthora nicotianae and Identification of Molecular Markers for the Oomycetes
Source: Front Microbiol. 2017 Aug 8;8:1484. doi: 10.3389/fmicb.2017.01484 (PMC5550686; doi:10.3389/fmicb.2017.01484)
Supplement: Supplementary file 8 [file Table_2.DOCX]

**Table S2.** Model test for 29 genes and the whole mitochondrial genome sequences

|  | Model | freqA | freqC | freqG | freqT | Kappa |
| --- | --- | --- | --- | --- | --- | --- |
| rpl16 | F81+G | 0.4465 | 0.1053 | 0.1429 | 0.3053 |  |
| rps11 | F81+G | 0.509 | 0.0972 | 0.0846 | 0.3093 |  |
| atp8 | HKY+G | 0.4465 | 0.0646 | 0.0398 | 0.449 | 3.1244(ti/tv=0.5853) |
| nad6 | HKY+G | 0.3886 | 0.0879 | 0.0828 | 0.4407 | 2.7625(ti/tv=0.7859) |
| rps2 | HKY+G | 0.4949 | 0.0691 | 0.0662 | 0.3698 | 2.5825(ti/tv=0.6113) |
| rps8 | HKY+G | 0.5038 | 0.0796 | 0.0717 | 0.345 | 2.7174(ti/tv=0.7071) |
| rps12 | HKY+G | 0.4614 | 0.1187 | 0.118 | 0.3018 | 1.4611(ti/tv=0.5414) |
| rps14 | HKY+G | 0.493 | 0.0849 | 0.0787 | 0.3435 | 2.5332(ti/tv=0.7027) |
| rpl14 | HKY+G | 0.4836 | 0.0927 | 0.0752 | 0.3486 | 2.8332(ti/tv=0.7890) |
| rps4 | HKY+G | 0.5219 | 0.0734 | 0.0684 | 0.3364 | 2.2907(ti/tv=0.5717) |
| rps10 | HKY+G | 0.5446 | 0.0784 | 0.0425 | 0.3345 | 1.9309(ti/tv=0.3933) |
| nad2 | HKY+I+G | 0.381 | 0.0738 | 0.0744 | 0.4708 | 2.3153(ti/tv=0.5889) |
| atp6 | GTR+G | 0.318 | 0.0878 | 0.1123 | 0.4819 |  |
| cob | GTR+G | 0.2953 | 0.1029 | 0.1514 | 0.4504 |  |
| cox1 | GTR+G | 0.2666 | 0.1138 | 0.1773 | 0.4424 |  |
| cox3 | GTR+G | 0.309 | 0.107 | 0.1504 | 0.4336 |  |
| nad1 | GTR+G | 0.2883 | 0.1081 | 0.1516 | 0.452 |  |
| nad3 | GTR+G | 0.2979 | 0.0673 | 0.1485 | 0.4862 |  |
| nad4L | GTR+G | 0.324 | 0.0612 | 0.0915 | 0.5235 |  |
| nad7 | GTR+G | 0.3686 | 0.1052 | 0.1732 | 0.3531 |  |
| nad9 | GTR+G | 0.4081 | 0.0872 | 0.1129 | 0.3919 |  |
| rpl2 | GTR+G | 0.4321 | 0.1145 | 0.1487 | 0.3047 |  |
| rpl6 | GTR+G | 0.4993 | 0.0501 | 0.0706 | 0.3799 |  |
| rps3 | GTR+G | 0.5067 | 0.0573 | 0.0729 | 0.3631 |  |
| atp1 | GTR+I+G | 0.3639 | 0.1097 | 0.182 | 0.3444 |  |
| cox2 | GTR+I+G | 0.3298 | 0.0999 | 0.1625 | 0.4078 |  |
| nad4 | GTR+I+G | 0.332 | 0.0774 | 0.1115 | 0.4791 |  |
| nad5 | GTR+I+G | 0.31 | 0.0869 | 0.1328 | 0.4703 |  |
| nad11 | GTR+I+G | 0.4467 | 0.0779 | 0.0984 | 0.377 |  |
| All | GTR+I+G | 0.3838 | 0.0851 | 0.1194 | 0.4118 |  |

|  | R(a)[AC] | R(b)[AG] | R(c)[AT] | R(d)[CG] | R(e)[CT] | R(f)[GT] | p-inv | gammashape |
| --- | --- | --- | --- | --- | --- | --- | --- | --- |
| rpl16 |  |  |  |  |  |  |  | 0.257 |
| rps11 |  |  |  |  |  |  |  | 0.914 |
| atp8 |  |  |  |  |  |  |  | 0.553 |
| nad6 |  |  |  |  |  |  |  | 0.409 |
| rps2 |  |  |  |  |  |  |  | 0.53 |
| rps8 |  |  |  |  |  |  |  | 0.399 |
| rps12 |  |  |  |  |  |  |  | 0.276 |
| rps14 |  |  |  |  |  |  |  | 0.252 |
| rpl14 |  |  |  |  |  |  |  | 0.291 |
| rps4 |  |  |  |  |  |  |  | 0.524 |
| rps10 |  |  |  |  |  |  |  | 0.546 |
| nad2 |  |  |  |  |  |  | 0.317 | 0.567 |
| atp6 | 2.5583 | 5.544 | 4.8472 | 2.113 | 8.0934 | 1 |  | 0.268 |
| cob | 3.5302 | 4.889 | 6.6595 | 1.877 | 10.2214 | 1 |  | 0.232 |
| cox1 | 3.5438 | 6.069 | 9.6138 | 1.599 | 12.1123 | 1 |  | 0.215 |
| cox3 | 10.233 | 12.17 | 14.195 | 3.248 | 22.3382 | 1 |  | 0.307 |
| nad1 | 3.6185 | 6.403 | 6.5053 | 3.176 | 9.8075 | 1 |  | 0.283 |
| nad3 | 28.852 | 34.56 | 16.99 | 2.818 | 73.4943 | 1 |  | 0.261 |
| nad4L | 11.845 | 2.993 | 2.1952 | 4.588 | 29.1231 | 1 |  | 0.195 |
| nad7 | 4.5884 | 3.483 | 4.4483 | 0.875 | 6.173 | 1 |  | 0.229 |
| nad9 | 6.2758 | 8.273 | 4.7049 | 12.18 | 10.9587 | 1 |  | 0.362 |
| rpl2 | 4.0301 | 2.208 | 3.7746 | 1.674 | 4.4555 | 1 |  | 0.318 |
| rpl6 | 3691.5 | 2802 | 731.93 | 2569 | 4326.58 | 1 |  | 0.388 |
| rps3 | 7.7838 | 7.012 | 2.3857 | 6.283 | 7.8871 | 1 |  | 0.491 |
| atp1 | 2.4628 | 3.186 | 6.0174 | 1.667 | 8.8058 | 1 | 0.437 | 0.662 |
| cox2 | 2.411 | 6.155 | 6.2831 | 1.246 | 13.7769 | 1 | 0.333 | 0.503 |
| nad4 | 4.5347 | 6.752 | 4.5554 | 3.871 | 11.2977 | 1 | 0.335 | 0.456 |
| nad5 | 4.2618 | 5.676 | 5.4798 | 2.076 | 7.9477 | 1 | 0.295 | 0.479 |
| nad11 | 5.2569 | 5.865 | 3.6693 | 5.814 | 6.0889 | 1 | 0.275 | 0.863 |
| All | 4.9499 | 5.164 | 4.0492 | 2.945 | 8.2151 | 1 | 0.331 | 0.668 |
